# Supplementary material for: Levels of Small Extracellular Vesicles Containing hERG-1 and Hsp47 as Potential Biomarkers for Cardiovascular Diseases
Source: Int J Mol Sci. 2024 Apr 30;25(9):4913. doi: 10.3390/ijms25094913 (PMC11084293; doi:10.3390/ijms25094913)
Supplement: Supplementary file 1 [file ijms-25-04913-s001.zip › ijms-2893255-supplementary.pdf]

Supplementary Materials:

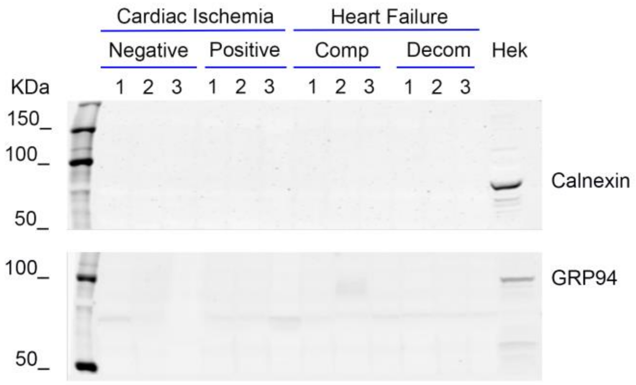

Figure S1. Calnexin and Grp94 were absent in the sEVs purified by ultracentrifugation. The presence of calnexin and Grp94 (MISEV2023. Protein content-based EV characterization as a negative control for sEVs [59]) was determined using Western blot, using a specific antibody for calnexin or Grp94. An equivalent amount of $1x10^9$ sEVs was considered for the analysis. Three samples were considered for each group of participants with different diagnoses of CVS. Comp: compensated; Decom: decompensated; Hek: homogenized from HEK293 cells growing under normal condition.

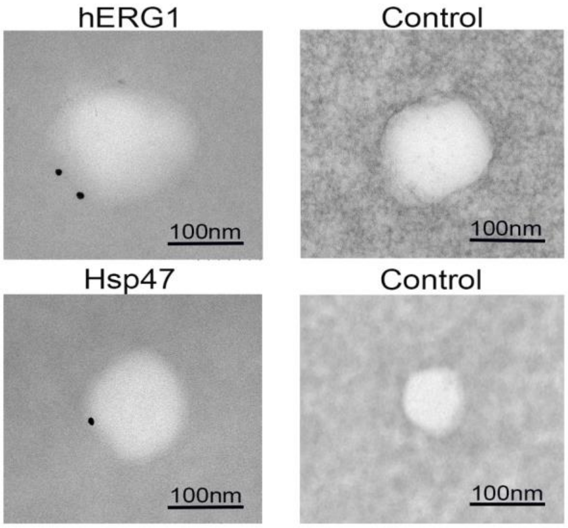

Figure S2. Transmission electron microscopy: sEVs purified from blood plasma by ultracentrifugation were incubated with anti-hERG1 or anti-Hsp47 antibodies. Then, they were incubated with a gold-conjugated secondary antibody (IgG-Au). The control consisted of sEVs incubated with only IgG-Au.

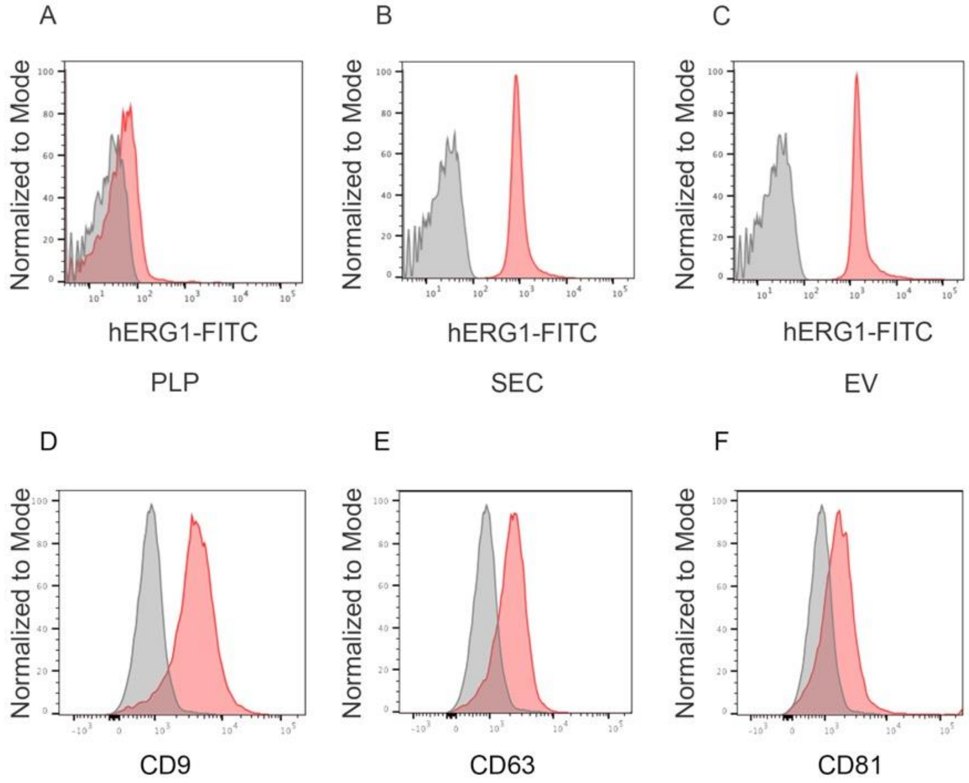

Figure S3. The expression of EV-hERG1 was detected using flow cytometry in blood plasma using one-step size exclusion chromatography sEVs purification. The presence of sEVs containing hERG1 (EV-hERG1) was determined using flow cytometry, using a specific antibody for hERG1 conjugated by FITC. An equivalent amount of 3.5×10^8 sEVs was considered for the analysis. Representative flow cytometry histograms of the presence of hERG1 using (A) platelet-free plasma (PFP); (B) size exclusion chromatography (SEC) sEVs purification; (C) extracellular vesicles (sEVs) purified using ultracentrifugation; (D) CD9 for extracellular vesicles (sEVs) purified using SEC; (E) CD63 for extracellular vesicles (sEVs) purified using SEC; (F) CD81 for extracellular vesicles (sEVs) purified using SEC. The grey region represents the basal signal, and the red region represents the hERG1 signal.

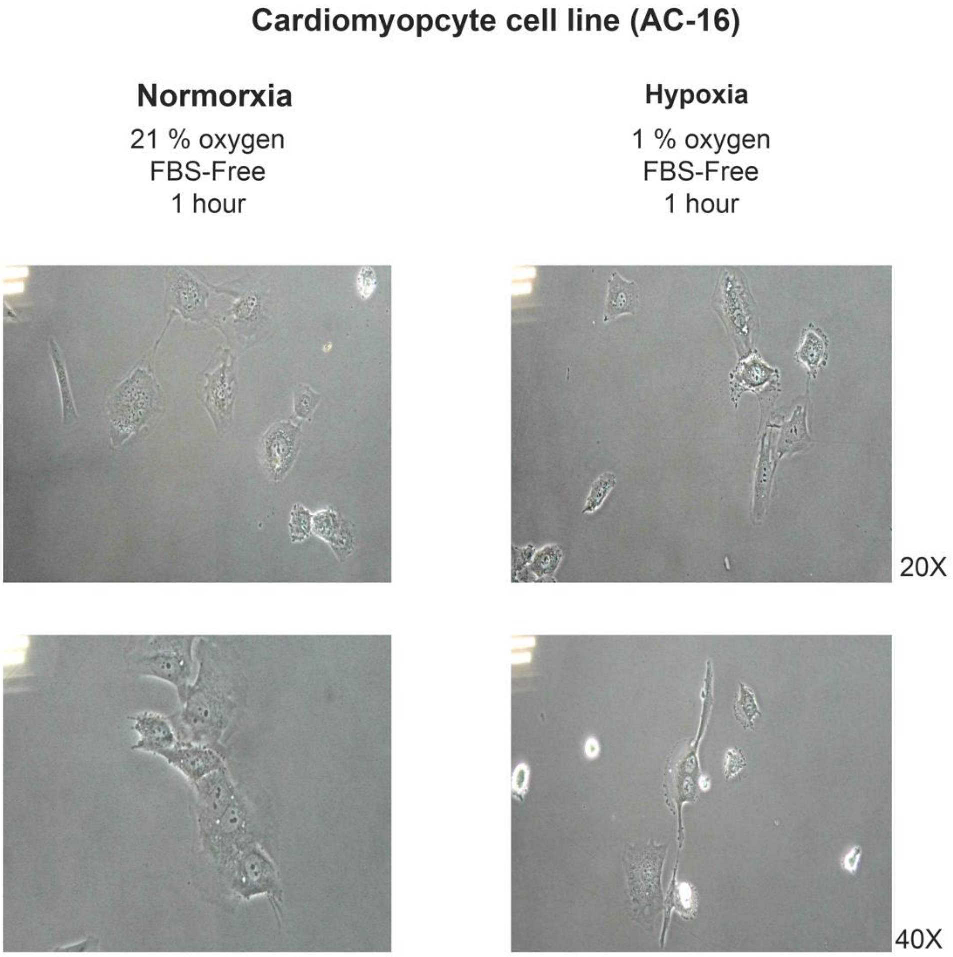

Figure S4. human cardiomyocyte cell line (AC-16) exposed to normoxia (21% oxygen) and hypoxia (1% oxygen) in an FBS-free culture medium for 1 hour.
